# Mushroom Poisoning in the Northeast of Iran; a Retrospective 6-Year Epidemiologic Study

**Published:** 2017-01-10

**Authors:** Bita Dadpour, Shahrad Tajoddini, Maliheh Rajabi, Reza Afshari

**Affiliations:** 1 Addiction Research Center, Faculty of Medicine, Mashhad University of Medical Sciences, Mashhad, Iran.; 2 Neuroscience Research Center, Institute of Neuropharmacology, Kerman University of Medical Sciences, Kerman, Iran.; 3 Medical Toxicology Research Centre, School of Medicine, Mashhad University of Medical Sciences, Mashhad, Iran.; 4 Environmental Health Services, BC Centre for Disease Control, Vancouver, Canada.

**Keywords:** Mushroom poisoning, epidemiology, mortality, toxicology, Iran

## Abstract

**Introduction:**

Toxic mushrooms are distributed across the globe with over 5000 species. Among them, 100 species are responsible for most of the cases of mushroom poisoning. This study aimed to evaluate the epidemiologic pattern of mushroom poisoning among patients referred to the main toxicology center of Mashhad province located in North-east of Iran.

**Method:**

This cross-sectional study was conducted on patients with final diagnosis of mushroom poisoning referred to the toxicology center of Mashhad, Iran, from February 2005 to 2011. Patients’ demographic characteristics, clinical presentations, laboratory findings, outcomes, and therapeutic measures were collected using a predesigned checklist and searching patient’s profile.

**Results:**

32 cases with the mean age of 24.6 ± 16.7 years were presented to the toxicology center following mushroom poisoning (59% female). Mushroom poisoning accounted for 0.1% of all admitted cases. The mean time elapsed from consumption to referral to poisoning department was 61.9 ± 49.4 hours. 19 (59%) cases were discharged with complete recovery, 7 (22%) expired, and 6 (19%) cases left hospital against medical advice. Mushroom poisoning mortality accounted for 1.5% of deceased cases in the studied center. There was significant relationship between mortality rate and higher values of INR (p = 0.035), PT (p = 0.011) and PTT (p = 0.003). Likewise, there was significant relationship between the need for mechanical ventilation and higher values of INR (p = 0.035), PT (p = 0.006) and PTT (p = 0.014). The relationships between the need for ICU admission, mechanical ventilation, and mortality rate with the rise of hepatic transaminases and serum bilirubin were not significant.

**Conclusion:**

Based on the findings, the prevalence of mushroom poisoning among patients referred to Mashhad toxicology center was very low (0.1%), but with a high mortality rate of 22%. Nausea and vomiting were the most common early symptoms of intoxication and higher values of coagulation profile were correlated with poor outcome.

## Introduction

The clinical picture caused by poisonous compounds in mushrooms is called mushroom poisoning or "mycetismus"(1). Toxic mushrooms are distributed across the globe with over 5000 species. Among them, 100 species are responsible for most of the cases of mushroom poisoning (2). Mushroom poisoning is responsible for 50 to 100 deaths per year in Western Europe. The frequency of mushroom poisoning is less common in the United States (3). Lethal dose of alpha-amanitin is reported to be 0.1 mg/kg. A single specimen of amanita phalloides may contain more than enough of this constituent to kill a man (4). The time interval between the ingestion of the mushrooms and the appearance of first symptoms is important for prognosis (1). This latency period is assessed as an independent prognostic factor (5). Exposures with late clinical manifestation are more toxic. Mushrooms with short incubation period (usually less than 6 hours) contain muscarine, coprin, ibotenic acid and psilocybin toxins in which clinical manifestation is mild and symptoms resolve in shorter period of time (1). 

Clinical manifestations following ingestion of toxic mushrooms are primarily gastrointestinal, which in some cases lead to hepatic, renal and nervous system damage and even death after a transient improvement phase (6). The Amatoxin is responsible for gastrointestinal symptoms as well as hepatic and renal failure (7). Hepatotoxic mushroom poisoning (due to Amanita, Lepiota and Galerina species) should be considered as a medical emergency, since an early diagnosis and immediate treatment are required for a successful outcome (6). This study aimed to evaluate the epidemiologic pattern of mushroom poisoning among patients referred to the main toxicology center of Mashhad province in North-east of Iran.

## Methods

***Study design***

This retrospective cross-sectional study was designed to evaluate the epidemiologic pattern of mushroom poisoning among patients referred to the toxicology center of Mashhad, Khorasan Razavi province, Iran, from February 2005 to 2011. All cases of intoxication in this province are referred to the studied center. The protocol of the study was approved by the ethic committee of Mashhad University of Medical Sciences and researchers adhered strictly to the Helsinki declarations and confidentiality of patients’ information.

***Participants***

All patients with final diagnosis of mushroom poisoning were enrolled using census sampling and without any sex or age limitation. Diagnosis was made based on history, clinical manifestation and epidemiologic data. No specific diagnostic laboratory tests were applied, as there were not available.

Latency period was defined as the time duration between the consumption of mushrooms and initiation of earliest gastrointestinal symptoms.

***Data gathering***

Patients’ demographic characteristics (sex, age, latency period, etc.), clinical presentations, laboratory findings (liver enzyme, coagulation profile, and bilirubin), outcome (death, discharge, need for intensive care unit (ICU) admission, and mechanical ventilation), and therapeutic measures (activated charcoal, fluids, penicillin G, and silymarin) were collected using a predesigned checklist and searching patient's profile. A trained toxicology resident was responsible for abstraction of data from patient’s profile.

***Statistical analysis***

SPSS (Statistical Package for Social Sciences) version 11.5 was used for statistical analysis. Data were reported as mean and standard deviation or frequency and percentage. Relationship between mortality rate and level of laboratory measures was calculated using chi-square or Fisher’s exact tests. A p-value of less than 0.05 was considered significant.

## Results

***Demographic***

During the study period, 32 cases with the mean age of 24.6 ± 16.7 (range: 6 - 73) years were presented to ED following mushroom poisoning (59% female, 62% resided in suburbs). Mushroom poisoning accounted for 0.1% of all admitted cases in the studied center. The mean time elapsed from consumption to referral to poisoning department was 61.9 ± 49.4 (range: 9 - 168) hours. Table 1 shows the frequency of patients’ sign and symptom at the admission time. The mean value of first recorded laboratory measures were as follows: aspartate aminotransferase (AST) 434 ± 947 (15 - 3812) IU/L, alanine aminotransferase (ALT) 534 ± 972 (10 - 3426) IU/L, total bilirubin 4.0 ± 6.3 (0.3 - 27.9) mg/dL, direct bilirubin 1.7 ± 2.5 (0.1 - 9.3) mg/dL, prothrombin time (PT) 24.6 ± 24.6 (11.9 - 121.0) seconds, partial thromboplastin time (PTT) 33.8 ± 12.5 (19.3 - 72.6) seconds, and international normalized ratio (INR) 2.5 ± 3.6 (0.9 -15.0).

***Treatment***

Penicillin G (1million units/Kg/day) and Silymarin (25-50 mg/Kg/day) were administered for 69% and 72% of poisoned cases, respectively. 25% of patients received activated charcoal and sorbitol prior to admission to the toxicology center.

***Outcome***

19 (59%) cases were discharged with complete recovery, 7 (22%) expired, and 6 (19%) cases left hospital against medical advice. Mushroom poisoning mortality accounted for 1.5% of deceased cases in the studied center. There was significant relationship between mortality rate and higher values of INR (p = 0.035), PT (p = 0.011) and PTT (p = 0.003). Likewise, there was significant relationship between the need for mechanical ventilation and higher values of INR (p = 0.035), PT (p = 0.006) and PTT (p = 0.014). There was significant relationship between the need for ICU admission and higher values of INR (P< 0.001). The relationships between the need for ICU admission, mechanical ventilation, and mortality rate with rise of hepatic transaminases and serum bilirubin were not significant.

**Table 1:** Frequency of patients’ sign and symptom at the admission time

**Complaint**	**Number (%)**
**Nausea**	28 (88)
**Vomiting**	24 (76)
**Vertigo**	16 (50)
**Loss of consciousness **	7 (22)
**Epigastric pain**	4 (13)
**Convulsion**	2 (6)
**Icter**	4 (13)

## Discussion

Based on the results of the present study, the prevalence of mushroom poisoning among patients referred to the main toxicology center of Mashhad province located in North east of Iran was very low (0.1%), but with a high mortality rate of 22%. Nausea and vomiting were the most common early symptoms of intoxication and higher values of coagulation profile were correlated with poor outcome. 

Nearly 10,000 species of mushrooms are identified in the world and approximately 50-100 species are considered as poisonous (8). About 90% of mushrooms that involved in exposures could not be identified (9). Amanita species (including A. phaloids, A. virosa and A. verna) , Gyromitra esculenta, and the Galerina species are three most common poisonous mushrooms (10). Amanita grows in many forest cover areas in Iran such as Mazandaran, Gilan and Azarbaijan (Forest Arasbaran) and is the most dangerous toxic mushroom in Iran and worldwide (11).

Mushroom poisoning was less frequent in our study in comparison to a study conducted in Turkey (3). The case-fatality rate, however, was higher. In Turkish study just 12 out of 143 patients died due to fulminant hepatic failure. Even lower mortality rate was reported from Texas (12).In contrast, much higher mortality rate has been observed from Plovdiv region of Bulgaria (13). This difference could be attributed to variation of mushroom species, different amount of ingested amatoxin or even individual susceptibility (1). Case finding could also be limited in some areas. Higher case-fatality rate in this study could be related to limited case reporting capabilities where just more dramatic cases are being referred. 

Despite widespread application of some empiric treatments including non FDA approved penicillin G, silymarin, no definite treatment has been introduced for mushroom poisoning to date (14). In this study, nonspecific antidotes including penicillin G, silymarin were administered in majority of cases in addition to conservative treatments. Penicillin G displaces amanitin from the binding to plasma protein and thus promoting its excretion and preventing its hepatic uptake and also enhances the elimination of the toxin from the kidneys (15). 

Although most mushroom poisonings are presented with gastrointestinal symptoms alone and cases are usually discharged with recovery, but liver function impairment could happen in some cases and may lead to life threatening consequences (16). 

Taking the need for mechanical ventilation, or ICU admission and death as determinants for poor prognosis in mushroom poisoning, impairment of coagulation tests (PT, PTT and INR) represented poor outcome in this study whereas increased transaminases (AST and ALT) played no role in prognosis, even though they increased to more than 10 fold of normal limits. Therefore, ordering of early and serial coagulation tests is recommended. 

Considering high mortality rate, all patients with the history of mushroom ingestion should be admitted; accordingly treated and followed up (17). Alpha amanitin levels should be checked where possible, if amanita poisoning is suspected. If laboratory detection of toxin is not available, history of mushroom ingestion, clinical manifestation and their trends could define mushroom poisoning (18). 

**Limitation**

As a retrospective investigation, we were required to extract information from medical records and some necessary data were not recorded properly. 

## Conclusion

Based on the results of the present study, the prevalence of mushroom poisoning among patients referred to the main toxicology center of Mashhad was very low (0.1%), but with a high mortality rate of 22%. Nausea and vomiting were the most common early symptoms of intoxication and higher values of coagulation profile were correlated with poor outcome. 
